# Optimising partner notification outcomes for bacterial sexually transmitted infections: a deliberative process and consensus, United Kingdom, 2019

**DOI:** 10.2807/1560-7917.ES.2022.27.3.2001895

**Published:** 2022-01-20

**Authors:** Sonali Wayal, Claudia S Estcourt, Catherine H Mercer, John Saunders, Nicola Low, Tamsin McKinnon, Merle Symonds, Jackie A Cassell

**Affiliations:** 1 Institute for Global Health, University College London, London, United Kingdom; 2 School of Health and Life Sciences, Glasgow Caledonian University, Glasgow, United Kingdom; 3 Institute of Social and Preventive Medicine, University of Bern, Bern, Switzerland; 4 Western Sussex Hospitals NHS Foundation Trust, Worthing, West Sussex, United Kingdom; 5 Brighton and Sussex Medical School, Falmer, East Sussex, United Kingdom

**Keywords:** Sexually Transmitted Infection, STI, partner notification, Outcome Measures, BASHH

## Abstract

Partner notification (PN) is an essential element of sexually transmitted infection (STI) control. It enables identification, treatment and advice for sexual contacts who may benefit from additional preventive interventions such as HIV pre- and post-exposure prophylaxis. PN is most effective in reducing STI transmission when it reaches individuals who are most likely to have an STI and to engage in sexual behaviour that facilitates STI transmission, including having multiple and/or new sex partners. Outcomes of PN practice need to be measurable in order to inform standards. They need to address all five stages in the cascade of care: elicitation of partners, establishing contactable partners, notification, testing and treatment. In the United Kingdom, established outcome measures cover only the first three stages and do not take into account the type of sexual partnership. We report an evidence-based process to develop new PN outcomes and inform standards of care. We undertook a systematic literature review, evaluation of published information on types of sexual partnership and a modified Delphi process to reach consensus. We propose six new PN outcome measures at five stages of the cascade, including stratification by sex partnership type. Our framework for PN outcome measurement has potential to contribute in other domains, including Covid-19 contact tracing.

## Background

Partner notification (PN) is a key strategy in sexually transmitted infection (STI) control. It entails identifying and informing sex partners of persons diagnosed with STI to facilitate their timely testing and treatment, to prevent reinfection of the person diagnosed (index patient) and onward transmission of infection [1]. Partner notification is an important way of identifying, and providing care to, individuals who are unaware they have an STI. It has a high yield of positive test results compared with both population screening and testing of symptomatic individuals in sexual health services. In addition, PN facilitates the identification of individuals who could benefit from preventive interventions such as HIV pre-exposure prophylaxis (PrEP) and post-exposure prophylaxis, hepatitis vaccination and behavioural advice.

**Figure 1:**
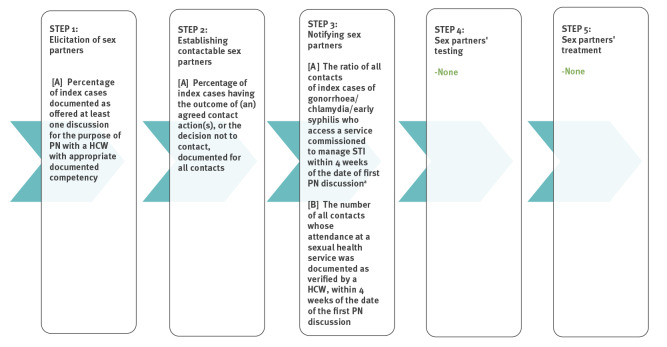
The partner notification cascade and outcome measures for evaluation of bacterial STI services, United Kingdom, 2019-

HCW: healthcare worker; PN: partner notification; STI: sexually transmitted infections.

^a^ Wording as published by BASHH.

The provision of PN, and monitoring of its performance, is key to improving the effectiveness of public health services for STI care. The process of PN can be described in a care cascade, with distinct stages and outcomes that can contribute to processes of service quality improvement. Figure 1 shows five steps: finding out from the infected person (index case) the sex partners who might have been exposed (elicitation), establishing those that can be contacted, notifying the partners, ensuring testing and ensuring treatment. At each step, there may be a reduction in the proportion of sex partners that is reached. Effective PN should retain as many index patients and partners as possible throughout the cascade.

Standards used to monitor PN outcomes should address all steps in the PN cascade and should consider key factors that affect PN outcomes. In the United Kingdom (UK), the British Association for Sexual Health and HIV (BASHH) has published standards for PN for bacterial STI, with auditable outcome measures, last updated in 2012 [2,3]. Figure 1 shows these established outcome measures for PN services, which cover only the first three stages of the cascade. Their partial coverage of the PN cascade limits our understanding of the effectiveness of PN services as it does not include outcome measures for processes involved in the testing and treatment of sex partners.

Services for the diagnosis and care of sexually transmitted infections, including partner notification, vary across Europe. In the UK, sexual health clinics provide much of this care, especially in urban areas, with primary care services providing some less specialised testing and care. Also, the access to STI services varies across Europe, with gynaecology, primary care and sexual health services having a varying role, as do their approaches to partner notification. While legal and policy frameworks are diverse, the principles of partner notification in STI control remain the same, requiring different approaches to data collection and evaluation [4].

In this Perspective article, we describe the process of evidence gathering, deliberation and consensus-building to address these gaps in current PN outcome measures and standards for bacterial STI in the UK, and to provide recommendations for future PN services internationally.

## The importance of partner type in partner notification outcomes

Sexual behaviours, including the type of sexual partnership(s) and the force of infection in the sexual network, influence individuals’ STI risk and their risk of onward STI transmission [5]. Partner notification is most effective in reducing STI transmission in a population if it reaches individuals whose sexual behaviours and/or partnerships increase their STI risk and/or who are at greater risk of onward STI transmission in their sexual networks.

Current PN outcome measures do not differentiate between different partner types. In the case of chlamydia infection, for example, the UK specifies as a standard that 0.6 contacts per index case with chlamydia should access sexual healthcare service within 4 weeks of the date of the first PN discussion. This standard is an average of outcomes for all sex partners, which does not take into account differences between sex partner types that affect STI prevention at the population level [6]. For example, a patient diagnosed with chlamydia may be highly motivated to undertake PN for an ongoing partnership, but less so for a partner with whom they have had sex only once. The latter type of partner could be considered a ‘high-value’ sex partner because they might be more likely to transmit infection to others yet less likely to receive treatment (Step 5 of the PN cascade) than an ongoing partner [6]. In this case, a clinic can achieve the performance standard, but it cannot assess the extent to which high-value sex partners have been reached.

Analysis of data from Britain’s National Survey of Sexual Attitudes and Lifestyles has shown that sexual partnership type is associated with the probability of a recent STI diagnosis [5]. This evidence supports the hypothesis that different approaches to PN, targeting different types of sex partners, could enhance its effectiveness and reduce STI transmission at a population level [7]. Although this insight is recognised informally in UK guidance, it has not yet been incorporated into PN outcome measures. For example, guidelines on how to take a sexual health history [8] recognise the relevance of partnership type, recommending that an index patient should be asked about their last sexual contact and any previous sex partners. Clinicians are asked to assess the type of partnership with sex partners (in informal terms such as live-in, regular, casual partner, etc.), duration of the relationship, and whether the partner could be contacted to facilitate PN [8]. However, there are no established definitions of partnership type, and they do not play a part in specifying outcome measures for PN.

## Development of updated partner notification outcome measures

A review and update of PN outcome measures and standards were commissioned by BASHH from the research team of the Limiting Undetected Sexually Transmitted Infections to RedUce Morbidity (LUSTRUM) programme. It is a 5-year programme of mixed methods research to improve PN methods, effectiveness and practice, with investigators from eight UK and one Swiss institution. The LUSTRUM study team includes experts in clinical sexual health medicine, health psychology, qualitative research, epidemiology, statistics, health economics and mathematical modelling and includes members of the National Surveys of Sexual Attitudes and Lifestyles (Natsal) study team. The update was required to address PN outcomes which could be used to specify expected standards of care, and how these could be differentiated by sex partnership type.

We undertook this update in three stages. Firstly, we performed two literature reviews to identify potential outcome measures for PN practice. We conducted a systematic review on MEDLINE building on a 2013 existing Cochrane review [9] and updating it until 18 December 2018, to identify outcome measures used in randomised controlled trials of PN for bacterial STI in high-income English-speaking settings [9]. Further details of the search strategy are given in the Supplement. We collated and reviewed all PN outcomes specified in PN guidelines in the following high-income English-speaking settings: Australasia (comprising Australia, New Zealand, neighbouring islands in the Pacific Ocean and Papua New Guinea), Canada, the UK and the United States.

Secondly, we used a classification of sex partner types, developed by researchers in the LUSTRUM programme, drawing on qualitative research and existing literature on partnership type [5,10]. The LUSTRUM team is already using the partnership classification in data collection and analysis of an RCT of PN [11]. The underpinning primary and secondary research describing the process of development is published elsewhere [12].

Thirdly, we undertook a modified Delphi process to present the findings to experts, discuss the results and reach a consensus on measurable PN outcomes covering all five stages of the PN cascade and taking into account partnership type [14]. In advance of these meetings, information on measurable PN outcomes was circulated to the experts in order to facilitate discussion. A first meeting, on 27 March 2019, brought together seven of the authors of this manuscript as a multidisciplinary group of internal experts from the LUSTRUM team to choose candidate PN auditable outcome measures from those identified during the literature review. This was followed by another meeting on 8 May 2019, in which these experts were joined by a further seven multidisciplinary external experts and stakeholders whose details are given in the Supplement. During this meeting, through a process of discussion and consensus building, a final set of optimal outcomes was chosen for final adoption into the BASHH PN national recommendations.

**Figure 2:**
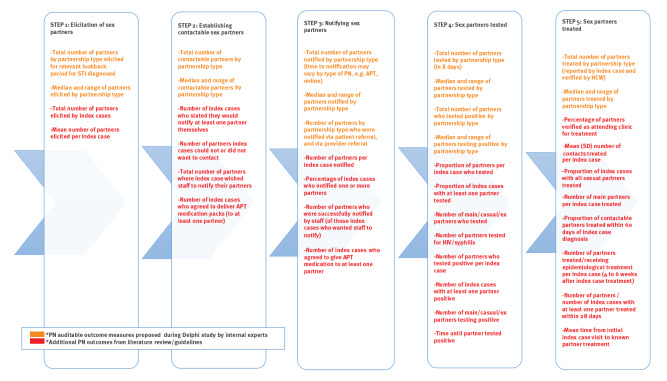
Partner notification processes and related outcome measures

APT: accelerated partner therapy; BASHH: British Association for Sexual Health and HIV; HCW: healthcare worker; PN: partner notification; SD: standard deviation; STI: sexually transmitted infections.

**Figure 3:**
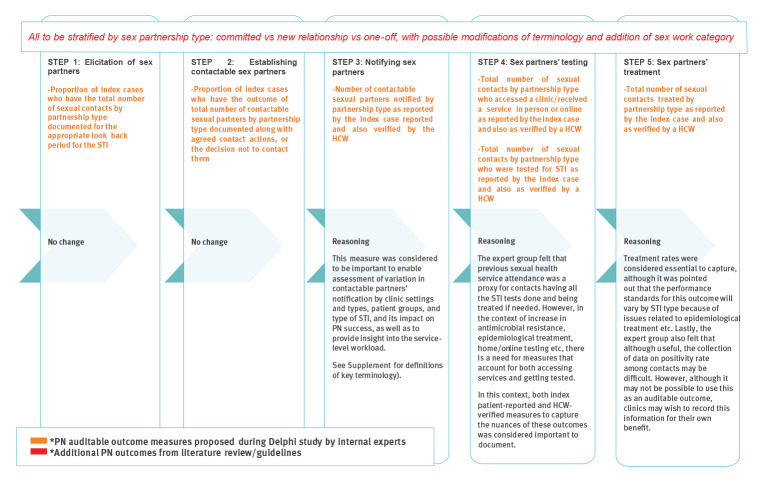
Consensus recommendation on partner notification auditable outcome measures

BASHH: British Association for Sexual Health and HIV; HCW: healthcare worker; PN: partner notification; STI: sexually transmitted infections;

Finally, we mapped all PN outcome measures for bacterial STI identified in the review of RCT onto the PN cascade shown in Figure 1 (see the Supplement for the findings of this mapping exercise). These were circulated to the internal experts group in advance of their meeting. For each step in the PN cascade, we proposed a list of candidate outcomes as highlighted in orange in Figure 2. This was circulated to the external experts group (attended also by internal experts) along with a brief report in advance of the second meeting. Discussion took place among external experts, with a particular focus on Steps 3–5 where there are major gaps in standards, where reasons for choices and prioritisation were explored in considerable details. This led to a consensus on a selection of proposed PN auditable outcome measures for all the five steps in the PN cascade, as shown in Figure 3.

## Development of recommendations on sex partnership categories within partner notification outcomes

The LUSTRUM team also developed five categories of sex partners drawing on empirical work within the research programme: 'committed, steady, occasional partner, new relationship and one-off partner'. It was highlighted that the LUSTRUM team had at that time demonstrated proof of feasibility by training staff members across 20 sexual health clinics to collect data using these partnership categories from index cases, subsequently collected in our RCT [15]. Experts discussed whether these categories have value to inform the targeting or approaches used in PN, and in evaluating outcomes.

The external experts concluded that overall, these five categories are useful and can potentially provide ways of reflecting on clinical practice and outcomes. They recommended that all PN outcome measures recommended should be stratified by partnership type. The external experts suggested a number of refinements to terminology, reflecting the complexities of clinical practice. These related to terminology for ongoing partnerships and how to determine when a previous partner becomes a new partner again.

## Summary of recommendations on outcome measures and sex partnership type

Figure 3 summarises the recommended outcome measures covering Steps 1–5 of the PN cascade, indicates which are new and which are already addressed in BASHH standards, and presents the accompanying recommendation that all should be stratified by partnership type. The external experts emphasised the need for sex partnership classifications to align with national guidance on how to take a sexual history, to integrate recommendations related to sex partner classification. This is required to ensure that such sex partnership data are routinely and uniformly collected by all services commissioned to provide sexual healthcare. A pilot of data collection was proposed in order to determine appropriate standards for the proposed PN outcome measures, to assess feasibility and ultimately align with BASHH PN standards. A pilot in the context of a national audit is planned in 2022. Given the range of settings providing sexual health services in the UK, an assessment of whether these outcomes can be collected in settings others than sexual health clinics, such as General Practice, was proposed.

## Conclusions

For effective evaluation of PN services for STI prevention, we need auditable outcome measures addressing each step of the PN cascade, which recognise the importance of partnership type. A more comprehensive and outcomes-focussed approach to auditing PN services will enable the cost-effective targeting of PN services and justify to funders the resources needed to do this. The need to establish and evaluate contact tracing processes at great scale and reach in the coronavirus disease (COVID-19) pandemic has sharply highlighted the need for meaningful and well understood PN outcome measures, which can be adapted to multiple infections and settings. The process of consensus established here in the context of bacterial STI, can contribute a framework for discussion well beyond the field of STI control.

## Ethical statement

No ethical approval was required for this process of literature review and expert consensus building.

## Supplementary Data

622000 Supplement
