# Supplementary material for: Optimising partner notification outcomes for bacterial sexually transmitted infections: a deliberative process and consensus, United Kingdom, 2019
Source: Euro Surveill. 2022 Jan 20;27(3):2001895. doi: 10.2807/1560-7917.ES.2022.27.3.2001895 (PMC8804665; doi:10.2807/1560-7917.ES.2022.27.3.2001895)
Supplement: Supplement [file 20-01895_CASSELL_Supplement.pdf]

Supplementary Material

Optimising partner notification outcomes. Wayal et al.

This supplementary material is hosted by “Eurosurveillance” as supporting information alongside the article “Optimising partner notification outcomes for bacterial sexually transmitted infections” on behalf of the authors who remain responsible for the accuracy and appropriateness of the content. The same standards for ethics, copyright, attributions and permissions as for the article apply. Supplements are not edited by “Eurosurveillance” and the journal is not responsible for the maintenance of any links or email addresses provided therein

Review method

Appendix: Systematic review method

We undertook a systematic search in Medline from 2011 to 18th December 2018, using the search strategy of a 2013 Cochrane review (1) modified by restriction of search terms to bacterial STIs of interest for this review (chlamydia, gonorrhoea, Trichomonas vaginalis, nonspecific urethritis and nongonococcal urethritis). We included all RCTs for bacterial STIs identified in the Cochrane review.

After deduplication, two reviewers independently screened the abstracts to determine the eligibility of studies for inclusion in the review and documented reasons for exclusion. Any RCTs that compared two or more methods of PN for bacterial STIs, and were conducted in European countries, United States of America (USA), Canada, Australia, and New Zealand were included in the review. Any discrepancies in the selection of studies were resolved by discussion between two researchers (SW and TM). A researcher (TM) extracted data on type of sex partnerships, PN outcome measures and their definitions used in eligible RCTs, using a data extraction form. All data was double-checked by a second researcher (SW). In communication with the Cochrane team which had also recently revised the search, a list of RCTs they had identified were compared with our results. Any eligible RCTs not picked up during our search were included.

1. Ferreira A, Young T, Mathews C, Zunza M, Low N. Strategies for partner notification for sexually transmitted infections, including HIV. Cochrane Database Syst Rev. 2013;10:CD002843.

Elicitation of sex partners (see App 4 for details of lookback period)

- Total number of partners elicited/ identified / reported by index patients
- Total number of partners elicited per index case
- Mean number of partners elicited per index

Establishing traceable sex partners

- Mean number of contactable partners
- Number of contact cards handed put by contact tracer per index patient
- Number of index patients who stated they would notify at least 1 partner themselves
- Total number of partners index wished study staff to notify
- Total number of partners with sufficient information to investigate
- Number of partners index could not or did not want to contact
- Number of index patients who requested assistance to notify at least one partner
- Number of partners located by contact tracer
- Number of study subjects who stated they would deliver medication to at least one partner.
- Number of study subjects who arranged to obtain medication for a partner from a commercial pharmacy
- Percentage of index patients who acknowledged another university student as a sexual partner in the last 6 weeks and agreed to provide names of partners

Notifying/contacting sex partners (see App 5)

- Total number of partners notified
- Percentage of partners notified
- Percentage of traceable partners contacted
- Number of partners per index case notified
- Percentage of indexes with 'all', 'some' or 'none of partners contacted
- Percentage of patients who notified 1 or more partners
- Percentage of index patients who reported seeing the partner
- Percentage of index patients reporting contacting 'all' or 'any' partners
- Proportion of index patients in the self-referral arm who had given each partner the information sheet listing the clinics where he could receive free treatment
- Percentage of index patients who had spoken to each partner about infection
- Percentage of index patients who had spoken to each partner about his need for treatment.
- Proprotion of index patients who told thier last partner they had sex with /current partner to take treatment
- Proportion of main/casual/ex partners notified
- Number of partners who were given/posted HSKs by the index
- Number of patients who agreed to give medication to at least one partner (EPT arm)
- Number of subjects that successfully obtained medication for thier partner from a commercial pharmacy
- Proportion of women in the PDPT arm who had given each partner the azithromycin/told partner to get treated/gave booklet
- Number of partners who study staff successfully notified (of those index patients wanted them to notify)

Sex partners presenting for care/testing (see App 6)

- Total number of partners presenting for care
- Proportion of partners preseting for care/ index patient
- Percentage of partners attending clinic within 8 or more days /within 1 week/1 month after index patient attending clinic
- Number of index patient referred to GUM clinic who did not attend
- Time taken until partner presented at clinic following index diagnosis

Sex partners tested

- Total number of partners tested
- Average number of partners tested per index case
- Proportion of partners per index patient who tested
- Proprotion of index patients with atleast one partner tested (laboratory results for HSKs)
- Number of main/casual/ex partners who tested
- Percentage of partners identified by index who presented for testing
- Time until partner testing (in days)
- Number of current partners who tested for HIV within 3 months
- Number of partners tested for HIV/syphilis

Sex partners diagnosed with STI (tested positive) (see App 7)

- Total number of sex partners who tested positive
- Number of partners who tested positive per index case
- Number of main/casual/ex partners testing positive
- Number of index patients with at least one partner positive
- Time until partner tested positive
- Number of partners who tested negative

Sex partners requiring treatment and time to it (see App 8)

- Total number of partners treated/ receiving epidemiological treatment
- Total number of partners treated (expressed as proportion of total number of partners)
- Total number of partners reported as "very likely" to have been treated or tested negative for STI (expressed as proportion of total number of partners)
- Total number of index patients who reported that all partners were 'very likely' to be treated or test negative (expressed as a proportion of total number of index patients)
- Mean (SD) contacts treated per index case
- Percentage of partners identified by index who presented for treatment
- Percentage of partners verified as attending clinic for treatment
- Proportion of index patients with all sexual partners treated (clinic records)
- Proportion of contactable partners treated within 60 days of index diagnosis
- Number of partners treated/receiving epidemiological treatment per index patient (4/6 weeks post randomisation/index treatment)
- Number of partners treated within 28 days
- Number of index patients with at least 1 partner treated in 28 days
- Number of partners very/somewhat likely/unlikely/unknown to have taken medication
- Mean time from initial index visit to known partner treatment.
- Percentage of index patients who reported checking to see whether partner was treated; that partner had informed them that they had taken medication; seeing partner taking the medication
- Total number of partners reported by index as havig received PDPT
- Percentage of index patients who, during interview following 2-week treatment, reported medication adherence
- Median time to treatment for partners with '≤30 days since sexual contact', '>30 days since sexual contact' and current partners between study arms
- Mean time from initial index visit to known male partner treatment
- Number of main partner per index treated

Sexual behaviour of index patients post treatment (see App 9)

- Number of days waited before resuming intercourse
- Percentage of index patients who reported that they had resumed sex with partner by the time of first follow-up
- Percentage of index patients who reported re-initiating sex with baseline partner
- Percentage of index who reported unprotected sex before partner treatment
- Percentage of patients who reported sex with partner unlikely to have been treated or test negative (expressed as proportion of total number of patients)
- Number of patients who do not anticipate having sex again with a partner.
- Percentage of index who reported unprotected sex with any partner
- Number (and percentage) of index patients who reported 1 or more acts of unprotected vaginal or anal intercourse in preceding 90 days, during follow-up interview 6 months after baseline
- Percentage of index patients who reported temporary abstinence from sexual intercourse during 14-day treatment period (during 2-weeks post enrolment interview)
- Percentage of index patients reporting vaginal sex and condom use in last 2 months during follow-up

Reinfection with STIs of index patients or test of cure uptake (see App 10)

- Number of index patients returning for test of cure within 14 days of initial clinic visit
- Percentage of index patients cured upon re-test 5-9 days post-treatment
- Proportion of index patients with a positive chlamydia test result 6 weeks-4 months after treatment for initial infection
- Proportion of men and women with a second episode of gonorrhoea and/or chlamydia occurring within 6 months of their initial diagnosis as ascertained through public health surveillance
- Reinfection rate for chlamydia measured through retesting (using PTKs) every 3 months over a 12 month period.
- Proportion of women who had one partner who was characterised as 'steady', and were re-diagnosed with chlamydia at follow-up
- Number of index patients with laboratory-confirmed chlamydia or gonorrhoea at the 3- or 6-month assessment (self-collected vaginal swab assay).
- Persistent or recurrent infection with gonorrhoea and/or chlamydia in patients 3 to 19 weeks after treatment/ 4 weeks after the initial clinic visit (with a window of 2-8 weeks)
- Re-infection rate of index patient (T. vaginalis culture) 4 weeks after the initial visit (with a window of 2-8 weeks)
- Chlamydia test positivity among women aged 14–25 y at the community level
- Incidence of reported gonorrhoea in women at the community level

Other

- Percentage of index patients receiving partner services following diagnosis, at population level
- Percentage of persons receiving PDPT (from their diagnosing clinicians/public health providers)
- Percentage of index patients reporting compliance with the PN intervention
- Adverse events in PDPT group.
- Number of patients who believe it is "very likely" an identified partner gave him/her the STI
- Returned confirmation slip of PDPT or returned PTK.
- Use of PDPT by medical providers
- Time interval between index treatment and dispensation of medication for partner
- Percentage of index patients who reported at initial PN interview that they were very or somewhat concerned that telling their partner of the chlamydial infection could result in a change in the relationship
- Percentage of index patients that reported being somewhat or very concerned that at least one sex partner would hurt or hit her if she told him that she had an STD or adverse events/instances of violence reported
- COST RELATED:
- Cost data based on clinic records of time spent on patient referrals with contact cards vs. contract referral
- Mean cost per partner traced (materials, incentives, counselling & telephone time) in a trial involving 3 arms of enhanced patient referral involving counselling, and/or contact cards and/or followup)
- Cost effectiveness (cost per woman; cost per partner treated; incremental cost per partner treated) in trial of patient referral vs booklet-enhanced patient referral vs EPT
- Cost per partner treated (clinic records) for trial comparing contract referral vs. provider referral vs. provider referral & field test for syphilis
- PN method leading to treatment (partner treatment either : a) verified by the partner calling the APTHotline or attending the APTPharmacy, b) reported by the index at a follow-up telephone assessment conducted by health advisers 4–6 weeks after index treatment).

Summary of partner notification processes (listed in white) and related outcomes (listed in black) used in randomised control trials comparing effectiveness of PN strategies

Definitions of key terminology

These are drawn from Sullivan et al. HIV partner notification for adults: definitions, outcomes, and standards published by BASHH, BHIVA, National AIDS Trust and Society of Sexual Health Advisers 2015, and available at http://www.missionmrcog.com/home/images/Library/BASHH_BHIVA_Guidelines/006_hiv_partner_notification_2015%20BHIVA.pdf

Contactable The full name, address and landline telephone or mobile phone number are ideal. A date of birth is useful to allow addresses to be obtained from the Patient Administration System (PAS), the Family Health Services Authority (FHSA) or the GP. Alternatives might include first name/ nickname/ profile name; email address; social media site; or school/college/university; place of work. However, the information available may be much more limited or may change throughout the PN process, such that an initially contactable person becomes un-contactable and vice versa. When reporting the outcomes, it is the final contact status that should be applied. However, it is the potential not the actual status that decides the category – a lack of response does not change the contact's status, e.g. someone who does not answer their phone remains contactable. As long as there is a means of contact, they should be classified as contactable –this could be as little as a first name and a mobile phone number.

Changing classification A contactable contact may have their classification changed if the details given prove to be incorrect/ineffective/ insufficient, and no further details are available from the index. An example would be a mobile number that is no longer active, but not one that is not answered. It would include a bounced email due to wrong address, but not 'inbox full'. It could also include 'profile deleted'. Similarly, a person may initially appear to be contactable because the index expects to see them, or find further contact details, or make contact through a third party. If, at follow-up interview, the index reports that efforts were unsuccessful, the contact should be re-classified as 'uncontactable'.

Outcomes The contact should only be classified as 'notified' if a patient or a health care worker has spoken to him/her, or there is evidence that a message sent via text, post, email or dating website has been received. Unacknowledged communication should not be recorded as a successful outcome e.g. unanswered text, no response to voice mail/letter

Name and organisation of internal experts who attended meeting, held on 27th March 2019, on auditable partner notification outcome measures for bacterial STI: shortlisting outcome measures from the proposed list
Professor Claudia S. Estcourt, Glasgow Caledonian University, UK
Professor Nicola Low, University of Bern, Switzerland
Professor Jackie A. Cassell, Brighton and Sussex Medical School, UK
Professor Catherine H. Mercer, University College London, UK
Dr Sonali Wayal, University College London, UK
John Saunders, Public Health England, Colindale, UK
Merle Symonds, Western Sussex Hospital, UK

Name and organisation of external and internal experts who attended meeting, held on 8th May 2019, on auditable partner notification outcome measures for bacterial STI: finalising outcome measures from the proposed list
Dr Ann Sullivan, BASHH clinical effectiveness group/audit representative
Ana Harb, GUMCAD representative, Public Health England
Martin Murchie, President of Sexual Health Advisors Association
Ceri Evans, Health Advisor, Chelsea and Westminster Hospital
Jonathan O'Sullivan, London Commissioner for Sexual Health

Hannelore Götz, Netherlands Public Health*
Jane Hocking, Australia Public Health/academic*
Professor Claudia S. Estcourt, Glasgow Caledonian University, UK
Professor Nicola Low, University of Bern, Switzerland
Professor Jackie A. Cassell, Brighton and Sussex Medical School, UK
Professor Catherine H. Mercer, University College London, UK
Dr Sonali Wayal, University College London, UK
John Saunders, Public Health England, Colindale, UK
Merle Symonds, Western Sussex Hospital, UK
*These experts provided further input to the draft partner notification outcome measures following this meeting.
